# Digital pathology and lipid droplet size as a key determinant of discrepancies between histology and MRI gradings in steatotic liver disease

**DOI:** 10.1007/s00330-025-11919-0

**Published:** 2025-08-08

**Authors:** David Marti-Aguado, Clara Alfaro-Cervello, Matías Fernández-Patón, Amadeo Ten-Esteve, Leonor Cerdá-Alberich, Ana Crespo, Irene Navarrete-Pérez, María Pilar Ballester, Alexandre Perez-Girbes, Cristina Montón, Judith Pérez-Rojas, Víctor Puglia, Antonio Ferrández, Victoria Aguilera, Desamparados Escudero-García, Salvador Benlloch, Ana Jimenez-Pastor, Ángel Alberich-Bayarri, Claude B. Sirlin, Luis Marti-Bonmati

**Affiliations:** 1 Digestive Disease Department, Clinic University Hospital, INCLIVA Health Research Institute, Valencia, Spain; 2 University of Valencia, Faculty of Medicine, Valencia, Spain; 3 Pathology Department, Clinic University Hospital, INCLIVA Health Research Institute, Valencia, Spain; 4 Biomedical Imaging Research Group (GIBI230) La Fe Health Research Institute, Valencia, Spain; 5 Digestive Disease Department, Hospital Arnau de Vilanova, Valencia, Spain; 6 Digestive Disease Department, La Fe University and Polytechnic Hospital, Valencia, Spain; 7 Radiology Department, La Fe University and Polytechnic Hospital, Valencia, Spain; 8 Pathology Department, La Fe University and Polytechnic Hospital, Valencia, Spain; 9 Pathology Department, Hospital Arnau de Vilanova, Valencia, Spain; 10 CIBERehd, Centro de Investigación Biomédica en Red en Enfermedades Hepáticas y Digestivas, Instituto de Salud Carlos III, Madrid, Spain; 11 Hepatology and Liver Transplantation Unit, La Fe University and Polytechnic Hospital, Valencia, Spain; 12 Universidad Cardenal Herrera-CEU Universities, Valencia, Spain; 13 Quantitative Imaging Biomarkers in Medicine, QUIBIM SL, Valencia, Spain; 14 Liver Imaging Group, Department of Radiology, University of California San Diego, La Jolla, CA USA

**Keywords:** Hepatic steatosis, Lipid droplets, Digital image analysis, Proton density fat fraction, Magnetic resonance imaging

## Abstract

**Background:**

Hepatic steatosis grades derived from magnetic resonance imaging proton density fat fraction (MRI-PDFF) might disagree with those determined by histology. We investigated whether the size distribution of lipid droplets (LDs) assessed with digital image analysis (DIA) explains the discrepancies between histology and MRI-PDFF.

**Materials and methods:**

Multicentric, prospective study of 355 patients with chronic liver disease, having paired biopsy and MRI. Using conventional microscopy, steatosis was graded by pathologists (S0-S3), based on the proportion of hepatocytes containing large LDs. MRI-PDFF graded steatosis using validated thresholds (PDFF-S1 ≥ 5.75%, PDFF-S2 ≥ 15.5%, PDFF-S3 ≥ 21.35%). DIA categorized LDs into tiny (< 1 μm^2^), small (1–100 μm^2^), and large (≥ 100 μm^2^) subtypes. Multivariable modeling was performed to identify predictors of discordances.

**Results:**

Histology- and PDFF-derived steatosis grades were discordant in 36%. Disagreement was associated with higher proportions of tiny and small LDs, and lower content of large LDs. Within histology-derived S0, disagreements were due to MRI overestimation having a higher content of tiny and small LDs. Within histology-derived S2-S3, disagreements were due to MRI underestimation, having a lower content of large and total LDs. Total LD proportionate area strongly correlated with MRI-PDFF (*r* = 0.89). DIA showed that as steatosis accumulates, the size of LDs progressively increases.

**Conclusion:**

Taking DIA as ground truth, the size distribution of LDs explains the discrepancies between histology and MRI-PDFF steatosis gradings. Compared to histology, MRI-PDFF overestimation is due to abundant tiny-small LDs, and underestimation is due to lower content of large LDs. These results indicate that MRI captures the whole spectrum of LD size, avoiding the conventional histology subjective assessment bias.

**Key Points:**

***Question*** *Can the size distribution of hepatic lipid droplets (LDs), determined with digital pathology, provide insights into the discordances between histology and MRI-PDFF steatosis grading?*

***Findings**** There is a 36% disagreement rate between histology and MRI-PDFF. Tiny and small LDs explain discordance, while large LDs favor concordance between both techniques*.

***Clinical relevance*** *MRI-PDFF captures the whole spectrum of LDs size beyond pathologists scoring system and strongly correlates with LDs total content avoiding conventional histology bias* *.*

**Graphical Abstract:**

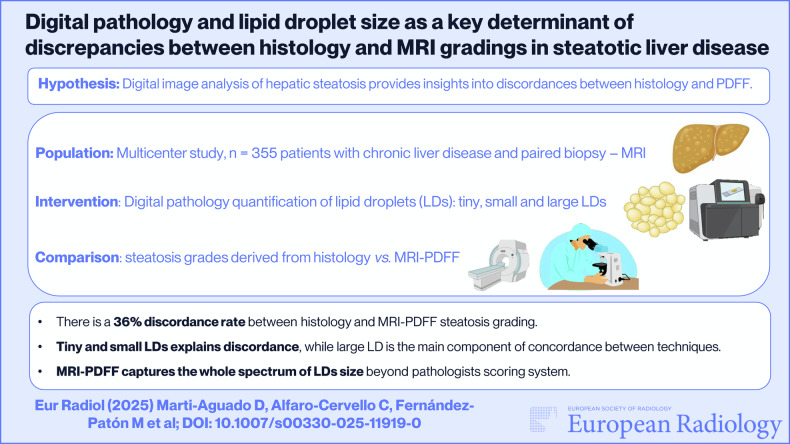

## Introduction

Steatotic liver disease is a global health issue [1]. The current standard for the assessment of hepatic steatosis remains histological evaluation. Using conventional microscopy, pathologists subjectively estimate the fraction of hepatocytes that contain large lipid droplets (LDs) displacing the nucleus to the cell periphery [2]. Potential pitfalls in the histological assessment of steatosis include small tissue sampling, inconsistency among pathologists, specimen processing procedures such as tissue fixatives inducing the collapse of LDs, and the influence of the staining method on the visualization of LDs [3–5]. Importantly, tiny and small LDs are routinely overlooked in histological grading, despite being increasingly recognized that these LDs are frequently present and might contribute to the overall burden of fat accumulation [6, 7].

MRI-proton density fat fraction (PDFF) is the most accurate, non-invasive method for quantification and grading of steatosis, being able to longitudinally assess and monitor patients under treatment [8, 9]. It has been suggested that MRI-PDFF could be more sensitive than biopsy to detect small changes in liver fat content [10]. A relative PDFF reduction ≥ 30% related to an intervention predicts a reduction of the metabolic dysfunction-associated steatotic liver disease (MASLD) activity score by ≥ 2 points, is associated with fibrosis regression and is accepted as an endpoint in phase-2 clinical trials [9]. Unfortunately, differences have been reported in steatosis grading derived by histology compared to MR-PDFF, having significant implications in clinical practice and trials assessing treatment response [11, 12]. To understand these differences and definitely validate MRI-PDFF as the reference for evaluating steatosis, large-scale studies are needed with precise tools capable of considering morphometric properties of steatosis on liver biopsy, such as digital pathology [8, 11].

Our hypothesis is that the size distribution of LDs might explain the observed discrepancies between histology and MRI-PDFF-derived steatosis grades. To measure LDs size, digital image analysis (DIA) was implemented, as this technique is increasingly recognized for a more granular and objective quantification of steatosis [13]. The use of digital pathology in liver transplantation and the allocation of donor organs has laid the groundwork for supporting DIA as the most accurate technique for quantifying LD content and size distribution [14, 15]. The primary objective was to assess the relationship between the size distribution of LDs and the discrepancies in steatosis grading as determined by histology and MRI-PDFF in a large, well-characterized cohort of patients with chronic liver disease (CLD).

## Material and methods

### Study design and participants

**Fig. 1:**
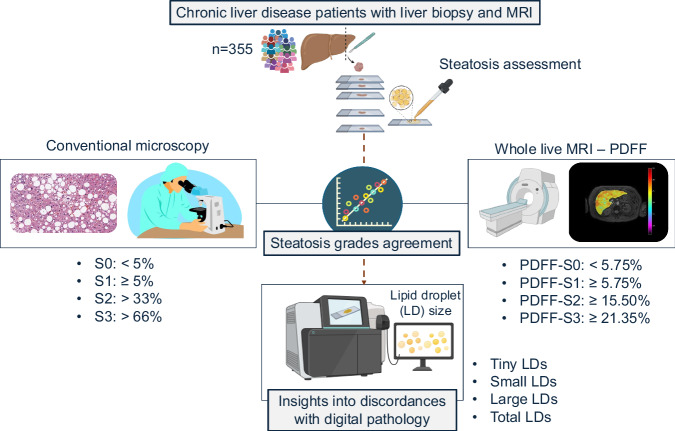
Graphical summary of the study design

This multicenter study was approved by the institutional review boards of the three participating hospitals (2016/209; 2017/0031/PI; 13/2019) and strictly followed the STARD reporting guidelines (Electronic Supplementary Material) [16]. A cross-sectional, prospective study with consecutive participant recruitment was performed between January 2017 and May 2024. Inclusion criteria were age ≥ 18 years, presence of CLD, liver biopsy performed for clinical care, and willingness to undergo an MRI examination within 40 days after biopsy. Exclusion criteria were contraindications to MRI, imaging artifacts, unsatisfactory biopsy with < 15 mm in length with < 6 portal tracts [3], and liver malignancy. After signing informed consent, all participants underwent MRI, vibration-controlled transient elastography (VCTE), and a complete biochemical profile (Supplementary Material). Figure 1 summarizes the study design.

### MRI examinations and analysis

Studies were centralized on a single 3-T MRI unit (Achieva TX, Philips Healthcare). All participants had a standard non-enhanced liver MRI reviewed by a radiologist to exclude patients with movement artifacts and focal liver lesions. A 2D multislice multiecho chemical shift-encoded gradient echo sequence was obtained in a single breath-hold acquisition with 12 echoes (TEs: 0.9-7.9, short echo spacing: 0.7 ms) and low flip angle (10°). The MRI scanning equipment and PDFF sequence parameters were the same for all patients. The calculation of PDFF was done using a single R2* water-fat signal model with six spectral peaks and combined magnitude-phase reconstruction optimized via least-squares fitting [17]. Whole-liver PDFF values were measured after automatic whole-liver segmentation (Quibim Precision 2.8-lab, Quibim SL) [18, 19]. Image analysts were blinded to histological data. For the purpose of this study, MRI-derived steatosis grades were classified as follows: PDFF-S0 < 5.75%; PDFF-S1 ≥ 5.75%; PDFF-S2 ≥ 15.5%; PDFF-S3 ≥ 21.35% [11].

### Histological evaluation

Percutaneous biopsies were obtained from the right lobe with a semiautomatic 16 G two-step needle. One biopsy sample was obtained per patient. Paraffin-embedded tissue sections were stained with hematoxylin-eosin and evaluated on conventional microscopy. All biopsies were centrally scored by two liver pathologists (> 15 years’ experience) blinded to imaging data. Histopathological analysis of liver tissue was jointly performed by the two dedicated pathologists, who reviewed the samples together and assigned scores based on mutual consensus. In the Banff consensus for steatosis assessment, a majority of liver pathologists define macrovesicular steatosis as only the large droplet subset [7]. As recommended by validated scoring systems (NASH-CRN and steatosis, activity, fibrosis score (SAF)), steatosis was graded as the frequency of hepatocytes with large LDs displacing the nucleus to the cell periphery (S0 < 5%; S1:5–33%; S2:34–66%; S3 > 66%) [20, 21]. Tiny and small LDs were disregarded for pathologist steatosis grading. Microvesicular steatosis (foci of hepatocytes with the cytoplasm being filled with tiny LDs without nuclear displacement) was scored as present or absent, not affecting the assigned steatosis grade [20, 21]. Other histological features were scored using the appropriate etiology-specific scoring systems based on the pathological diagnosis (Supplementary Material).

### Digital pathology

**Fig. 2:**
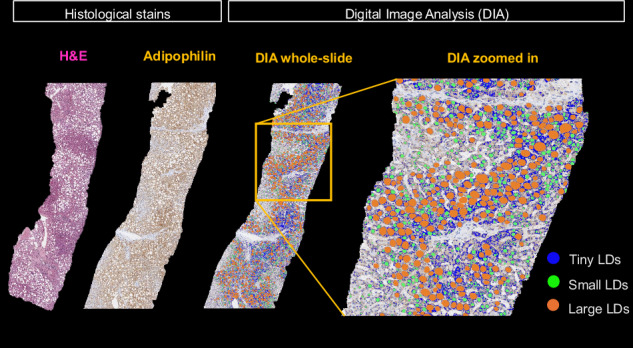
Digital image analysis (DIA) workflow. The two left images show the differences between tissue sections stained with hematoxylin-eosin (H&E) and adipophilin immunohistochemistry. Pathologists graded steatosis using H&E tissue sections. The two right images illustrate DIA using scanned adipophilin tissue sections. Different sizes of lipid droplets (LDs) were quantified

Additional sections were stained with adipophilin immunohistochemistry for DIA as previously described (Supplementary Material) [22]. DIA-derived metrics were expressed as the percentage of the lipid segmented ovoid or circular area relative to the total scanned liver tissue area (Fig. 2). LDs were classified into the following morphometric categories:

- Tiny LD: diffuse, faint, foamy appearance. This category represents the microvesicular droplets < 1 μm^2^ within the hepatocyte cytoplasm [6, 14, 23]. The vesicle size to define microvesicular steatosis was selected based on the Banff Liver Working Group recommendations [14]. Tiny LDs are often not discernible using light microscopy and usually require a fat stain to confirm [24].
- Small LD: vacuoles 1–100 μm^2^ surrounded by adipophilin stain. This category represents fat droplets occupying less than one-half of the hepatocyte without displacing the nuclei. Small LDs are not considered relevant in the overall assessment of steatosis by pathologists [7, 14].
- Large LD: vacuoles ≥ 100 μm^2^ surrounded by adipophilin stain, representing the macrovesicular droplets occupying more than one-half of the hepatocyte and pushing the nuclei to the cell periphery. The 100 μm^2^ threshold to distinguish small and large LDs was selected as the size required by most LDs to displace the nucleus from the center of the cytoplasm (Supplementary Material) [25].
- Total LD: as the ratio between the sum of tiny, small and large LDs, to the total tissue area.

### Endpoints

The primary endpoint was discordance between histology and MRI-PDFF-derived steatosis grades, defined as a difference of ≥ 1 grade. Considering DIA as the ground truth provides insights into discordances between histology and MRI. The secondary endpoint was the correlation between fat content assessed by conventional histology, MRI-PDFF, and the DIA-derived tiny, small, large and total LDs proportions.

### Statistical analysis

Sample size calculations accounted for an anticipated steatosis prevalence of 60% and a histology-MRI discordance rate of 50% [11, 12, 18]. With a significance level (*α*) of 0.05, power (1-β) of 0.80, and an effect size (*d*) of 0.05, an estimated sample of 369 participants was required [26]. Data was analyzed using SPSS V25.0 software package (IBM). All tests were two-sided, with *p* < 0.05 considered statistically significant.

#### Primary endpoint analysis

Differences between concordant and discordant groups were evaluated using the independent t-test or Mann–Whitney *U*-test for continuous variables and the chi-square or Fisher’s exact tests for categorical variables. Comparisons between paired distributions were assessed with the Wilcoxon matched pairs test, stratified by histological grades. Multivariable logistic regression analysis was performed to assess the independent predictors of discordance between histology and MRI-PDFF. We considered as candidate predictors: quality of biopsy specimens (length, portal tracts); conventional histology-derived features (microvesicular steatosis, significant fibrosis, inflammation grade); DIA-derived LDs proportionate areas; and PDFF closeness to established MRI-derived grade cutoffs (for example, both PDFF values of 3% and 18.25% would have a scalar distance of 2.75% to their closest grade thresholds of 5.75% and 15.5%, respectively) [11]. The multivariable model incorporated the variables with *p* < 0.05 from univariable logistic regression analysis, with adjustment for age and sex to estimate the odds ratios (OR) with 95% confidence intervals (95% CI).

#### Secondary endpoint analysis

Linear regression analysis was performed to determine the linearity and agreement between DIA-derived LD proportionate area as ground truth vs. histology-derived steatosis grades (Spearman rank correlation (*ρ*)) and vs. MRI-PDFF-derived fat content (Pearson’s correlation coefficient (*r*)).

## Results

### Patients characteristics

After applying exclusion criteria to the 429 screened participants, the final study sample included 355 patients (Supplementary Fig. 1). The most common etiology was MASLD and the median time interval between biopsy and MRI was 19 (14-28) days. The clinical characteristics, laboratory markers, and histological data of the participants are depicted in Table 1. The remaining conventional histological features of biopsy specimens are summarized in Supplementary Table 1.

**Table 1:** Baseline population descriptors including histological findings and imaging data

Characteristic	Overall sample
**Clinical**	
Sex (female)	209 (59%)
Age (years)	55 (47–63)
Smoking status (current/former)	87 (25%)
BMI (kg/m^2^)	27.9 ± 5.1
Liver disease etiology:	
• MASLD	219 (62%)
• MetALD	32 (9%)
• Autoimmune hepatitis	84 (24%)
• Viral hepatitis	8 (2%)
• Others	12 (3%)
Metabolic traits:	
• Obesity	115 (32%)
• Arterial hypertension	137 (39%)
• Diabetes mellitus	93 (26%)
• Dyslipidemia	175 (49%)
• Hypothyroidism	55 (15%)
VCTE—CAP (dB/m)	273 ± 65
VCTE—LSM (kPa)	9.9 ± 7.5
**Laboratory**	
Glucose (mg/dL)	98 (88–114)
Platelet count (×10^9^/L)	229 ± 71
Creatinine (mg/dL)	0.8 ± 0.2
ALT (U/L)	53 (35–89)
AST (U/L)	42 (31–67)
GGT (U/L)	89 (47–188)
Total bilirubin (mg/dL)	0.6 (0.5–0.8)
Albumin (g/dL)	4.4 (4.2–4.6)
Ferritin (mg/mL)	106 (44–234)
Triglycerides (mg/dL)	112 (78–170)
Total cholesterol (mg/dL)	190 ± 42
Low-density lipoprotein (mg/dL)	115 ± 35
**Histological**	
Macrovesicular steatosis grade:	
• Grade S0	169 (47%)
• Grade S1	69 (20%)
• Grade S2	52 (15%)
• Grade S3	65 (18%)
Microvesicular steatosis	149 (42%)
Digital pathology:	
• Tiny LD proportionate area (%)	5.7 ± 3.6%
• Small LD proportionate area (%)	0.6 ± 0.6%
• Large LD proportionate area (%)	2.4 ± 3.8%
• Total LD proportionate area (%)	8.7 ± 7.2%
**MRI**	
PDFF (%)	8.5 ± 7.2%
PDFF-derived steatosis grade:	
• Grade PDFF-S0	190 (53%)
• Grade PDFF-S1	106 (30%)
• Grade PDFF-S2	32 (9%)
• Grade PDFF-S3	27 (8%)

Unless otherwise specified, data are numbers of participants (*n* = 355), with percentages in parentheses. Data are reported as means  standard deviations when normally distributed and medians with interquartile ranges when the distribution is skewed

*ALT* alanine aminotransferase, *AST* aspartate aminotransferase, *BMI* body mass index, *CAP* controlled attenuation parameter, *GGT* g-glutamyl transferase, *LDs* lipid droplets, *LSM* liver stiffness measurement, *MASLD* metabolic dysfunction-associated steatotic liver disease, *MetALD* MASLD with increased alcohol intake, *MRI* magnetic resonance imaging, *PDFF* proton density fat fraction, *VCTE* vibration-controlled transient elastography

The distribution of histology-derived steatosis grades was S0 *n *= 169 (47%), S1 *n* = 69 (20%), S2 *n *= 52 (15%), and S3 *n* = 65 (18%). The distribution of MRI-PDFF-derived steatosis grades was PDFF-S0 *n* = 190 (53%), PDFF-S1 *n* = 106 (30%), PDFF-S2 *n* = 32 (9%), and PDFF-S3 *n* = 27 (8%). Mean PDFF was 8.5 ± 7.2%, ranging from 1.5%–38.5%.

### DIA assessment of lipid droplet size

**Fig. 3:**
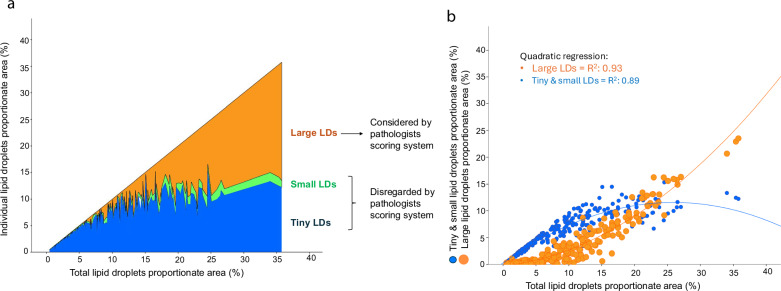
Distribution of different lipid droplets (LDs) size as a function of total LD proportionate area determined with digital pathology. **a** Stacked area chart showing the variation of LDs size with increasing total fat content. As fat accumulates, the size of LDs progressively increases. **b** Scatterplot of total LD proportionate area categorized in tiny + small (blue dots) and large droplets (orange dots). Quadratic regression was applied as a curved shape is seen with tiny-small LDs tending concave downwards and large LD tending concave upwards. *R*^2^ coefficient of determination is shown for each curve

Mean total LD proportionate area was 8.7 ± 7.2%, ranging from 0.5%–35.8%. Table 1 shows the DIA-derived LD size distribution. Among LDs categories, tiny LD accounted for the highest proportionate area, followed by large LD. As the DIA-determined total fat content increased, a shift in LDs size distribution was observed: patients with low fat content predominantly had tiny and small LDs, whereas large LD were more prevalent in those with higher fat content (Fig. 3). Notably, tiny LDs, which are not considered by pathologists’ grading, predominated in patients with less than 5% total LD content.

**Fig. 4:**
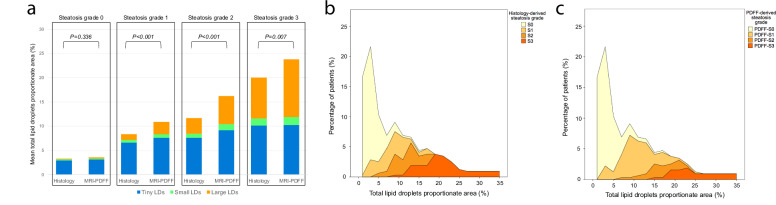
Distribution of lipid droplets (LDs) proportionate area derived by digital image analysis. **a** Stacked column bar plot showing the distribution of different LD sizes (tiny, small and large) for histology-derived and PDFF-derived steatosis grades. The total height of the column represents the total LD proportionate area. **b** Histogram showing total LDs proportionate area stratified by histology-determined steatosis grades (S0-S3). **c** Histogram showing total LDs proportionate area stratified by MRI-PDFF-determined steatosis grades (PDFF-S0–PDFF-S3). Histogram frequency represents the percentage of observations

The distribution of LDs proportionate areas across steatosis grades determined by histology and MRI-PDFF shows significant lower proportions of total LDs in histology-derived grades S1-S3 compared to their MRI-PDFF counterparts (Fig. 4 and Supplementary Table 2).

### Primary endpoint: disagreements between histology and MRI-PDFF

**Fig. 5:**
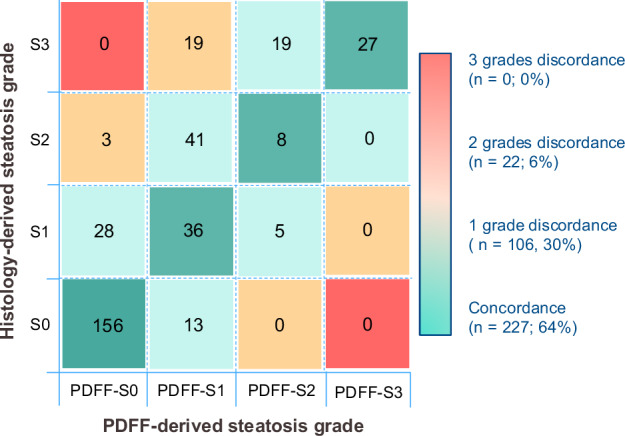
Overview of the distribution of hepatic steatosis grades and the discordances between histology and MRI-PDFF. Number of patients distributed across steatosis grade derived by histology (rows) and MRI-PDFF (columns)

There were statistically significant differences (*p* < 0.01) in the distribution of steatosis grades derived by histology and MRI-PDFF (Supplementary Fig. 2). MRI-PDFF classified more patients as grades S0 and S1, while histology classified more patients as grades S2 and S3. Overall, discordances were observed in 36% of cases (Fig. 5). Most discordant cases (*n* = 105) differed by 1 grade, while only 22 had a disagreement of ≥ 2 grades difference. Discordances predominantly affected patients with histology-derived steatosis grades S1, S2 and S3 (Supplementary Fig. 3). Among histology-derived S1, there was a 49% MRI-PDFF disagreement, mainly due to lower MRI grading. Among histology-derived S2 and S3, MRI-PDFF underestimated steatosis in 85% of S2, and 58% of S3 cases, consistently attributed to lower MRI grading.

Univariable and multivariable analyses are shown in Table 2. Tiny and small LDs proportionate areas were identified as independent predictors of discordances, while large LDs were independently associated with a lower risk of disagreement. A high content of tiny-small LDs favors discordances between histology and MRI-PDFF gradings, and a high content of large LDs favors concordance, probably related to what pathologists’ disregard and consider in their scoring system. The PDFF distance to the closest threshold was also independently associated with a lower risk of disagreement (Supplementary Fig. 4). In the univariable analysis, conventional histology-determined microsteatosis and significant fibrosis correlated with discordances, but only the presence of microsteatosis remained as an independent predictor of disagreement in the multivariable analysis. Interaction terms were tested between fibrosis stage and LD size, with no interaction effect between the two. Clinical and analytical data distribution among discordant cases is shown in Supplementary Table 3.

**Table 2:** Univariable and multivariable logistic regression analysis to evaluate predictive factors associated with discordance between histology-derived and MRI-PDFF-derived steatosis grade

Characteristic	Univariable analysis		Multivariable analysis^a^	
	OR (95% CI)	*p*-value	OR (95% CI)	*p-*value
Age (years)	1.03 (1.01–1.05)	0.002	1.02 (0.99–1.05)	0.128
Sex (male)	1.56 (1.00–2.41)	0.049	1.62 (0.92–2.85)	0.096
PDFF distance to closest threshold (%)	0.66 (0.55–0.80)	< 0.001	0.71 (0.58–0.86)	0.001
Biopsy length (mm)	1.10 (0.78–1.54)	0.590	-	-
Portal tracts (n°)	1.04 (0.99–1.10)	0.100	-	-
Microsteatosis	5.19 (3.24–8.29)	< 0.001	2.34 (1.20–4.56)	0.013
Inflammation grade ≥ 2	0.90 (0.55–1.49)	0.693	-	-
Significant fibrosis (F2-4)	2.57 (1.64–4.01)	< 0.001	1.25 (0.69–2.25)	0.460
Tiny LD (%)	1.29 (1.20–1.39)	< 0.001	1.20 (1.07–1.35)	0.002
Small LD (%)	3.27 (2.12–5.04)	< 0.001	2.60 (1.31–5.19)	0.007
Large LD (%)	1.09 (1.03–1.16)	0.006	0.88 (0.79–0.99)	0.047
Total LD (%)	1.10 (1.06–1.14)	< 0.001	-	-

*LD* lipid droplet, *MASLD* metabolic dysfunction-associated steatotic liver disease

^a^ Multivariable logistic regression analysis was performed. Along with age and sex, significant univariable predictors were entered into the multivariable analysis. Total LD was removed from the multivariable model due to multicollinearity with large LD

**Fig. 6:**
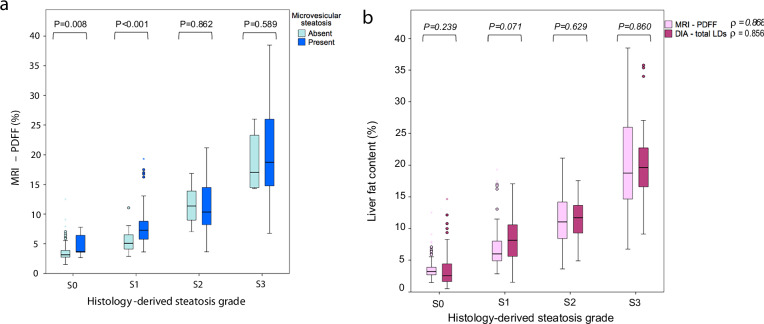
Box and whisker plots of (**a**) MRI-PDFF (%) stratified by presence (dark blue boxes) or absence (light blue boxes) of microvesicular steatosis; vs. histology-derived steatosis grades (S0-S3); and (**b**) liver fat content (%) stratified by MRI-PDFF (pink boxes) and digital image analysis-DIA (purple boxes), vs. histology-derived steatosis grades (S0-S3). A similar strength of correlation (Spearman rank coefficient (*ρ*)) was seen between histology-derived steatosis grade with MRI-PDFF and DIA. Comparisons between paired distributions were assessed with the Wilcoxon matched pairs test stratified according to histological steatosis grades

Table 3 summarizes LDs size distributions across concordant vs. discordant cases for each histological steatosis grade. Within histology-derived S0, MRI-PDFF disagreement was mainly associated with a higher content of tiny and small LDs. MRI-PDFF was significantly higher when pathologists determined the presence of microsteatosis on conventional histology (Fig. 6a and Supplementary Table 4). Among histology-derived S2 and S3 grades, MRI-PDFF disagreement was associated with a lower content of large and total LDs. Results of histology-derived S1 grade are discussed in the Supplementary Material.

**Table 3:** Mean proportionate areas of LDs by size in concordance vs discordance groups and stratified by histological-derived steatosis grades

Digital image analysis	Concordance	Discordance	*p*-value
**Histological steatosis grade S0 (*n*** = **169)**			
• Tiny LD (%)	2.7 ± 1.8%	5.7 ± 2.9%	< 0.001^a^
• Small LD (%)	0.2 ± 0.1%	0.3 ± 0.2%	0.017^a^
• Large LD (%)	0.1 ± 0.4%	0.5 ± 0.4%	0.019^a^
• Total LDs (%)	3.1 ± 2.1%	6.5 ± 3.1%	0.008^a^
**Histological steatosis grade S1 (*n*** = **69)**			
• Tiny LD (%)	7.5 ± 2.7%	5.3 ± 2.8%	0.004^a^
• Small LD (%)	0.6 ± 0.4%	0.5 ± 0.5%	0.768
• Large LD (%)	1.2 ± 1.2%	1.2 ± 1.7%	0.845
• Total LDs (%)	9.3 ± 3.1%	6.9 ± 4.3%	0.025^a^
**Histological steatosis grade S2 (*n*** = **52)**			
• Tiny LD (%)	7.6 ± 1.6%	7.6 ± 2.1%	0.941
• Small LD (%)	0.9 ± 0.4%	0.8 ± 0.4%	0.819
• Large LD (%)	5.6 ± 2.5%	2.9 ± 1.7%	0.001^a^
• Total LDs (%)	14.0 ± 2.6%	11.3 ± 3.1%	0.050
**Histological steatosis grade S3 (*n*** = **65)**			
• Tiny LD (%)	10.3 ± 1.7%	10.0 ± 2.1%	0.654
• Small LD (%)	1.7 ± 0.7%	1.4 ± 0.5%	0.080
• Large LD (%)	11.9 ± 4.3%	5.9 ± 2.6%	< 0.001^a^
• Total LDs (%)	23.4 ± 5.1%	17.3 ± 3.8%	< 0.001^a^

Unless otherwise specified, data are means ± standard deviations. Differences in characteristics across groups were tested using an independent *t*-test for continuous variables

The distribution of the data is presented for concordant vs. discordant groups, stratified by grades of conventional histological-derived steatosis (S0-S3)

*LD* lipid droplet

^a^ Indicates statistically significant differences (*p*  < 0.05)

### Secondary endpoint: correlations with DIA

Significant correlations were observed between histological steatosis grades and DIA-derived LDs size distributions (Supplementary Fig.  5). The strongest correlation was seen between large LD and histology-derived steatosis grades (*ρ* = 0.87; *p* < 0.01). Regarding MRI-PDFF, correlations progressively strengthened from tiny to total LDs (Supplementary Fig. 6). The strongest correlation was observed between total LDs and MRI-PDFF (*r *= 0.89; *p* < 0.01).

Finally, when stratified by histology-derived grades, a comparable stepwise increase was observed for liver fat content determined by MRI-PDFF and the proportionate area of LDs determined by DIA (Fig. 6b and Supplementary Table 5).

## Discussion

This large multicenter cohort of 355 patients with CLD evaluated with histology, DIA and MRI, revealed several key findings. LDs increase in size as the steatosis burden rises, likely reflecting the fusion of tiny to small droplets into larger ones. There is a 36% disagreement between steatosis grades from pathologists and MRI-PDFF. Compared to histology-derived grades, the disagreement mainly involves higher MRI-PDFF in S0 and lower MRI-PDFF in S1-S3 grades. Discordant cases with histology-derived S0 have a higher content of tiny-small LDs, as MRI-PDFF captures the entire spectrum of LD size. A lower content of large and total LDs among discordant cases with histology-derived S2-S3 suggests that the pathologist’s assessment overestimates steatosis. Finally, MRI-PDFF shows a strong correlation with DIA, regardless of LD size distribution.

Gold standard tests are not perfect and should be challenged and superseded when appropriate. Despite MRI-PDFF's high accuracy [9–11], inherent differences between histology and MRI entail implications for steatosis estimation. First, the NASH-CRN validated MRI-PDFF cutoffs for classifying steatosis grades are significantly different from those validated using histology [27]. Second, the corresponding values of liver fat content measured by MRI-PDFF are markedly lower as compared with the near-continuous histology-derived score (100% histological steatosis corresponded to 33% PDFF) [11, 27]. Guidelines highlight an unmet need to understand these differences by using DIA as the ground truth [8]. To fill this knowledge gap, we evaluated the size of LDs on biopsy using a precise DIA approach. The premise behind our hypothesis is that conventional histology disregards tiny and small LDs, while MRI-PDFF does not.

In line with previous MASLD studies [12], our CLD cohort validates the relevant disagreements between histology and MRI-PDFF grading. However, our concordance rate was higher than in complementary studies comparing fibrosis stage distribution between VCTE and MRE [28]. Our results support that discordant cases have features of more advanced disease [12]. Beyond these associations, we explored with DIA a deeper explanation of the disagreements between the two methods. Among individuals with histology-derived S0, MRI-PDFF upgraded 7% of them. These cases, compared to the concordant ones, had a higher content of tiny-small LDs. These LDs are disregarded by pathologists grading [7, 20, 21], but they are indeed captured by MRI, as also demonstrated by a higher PDFF when pathologists report that microsteatosis is present. Among individuals with histology-derived S2 and S3, MRI-PDFF downgraded 85% and 58%, respectively. These cases, compared to the concordant ones, had a lower content of large and total LDs, suggesting an overestimate by the pathologists´ assessment. Estimated liver fat content by pathologists systematically exceeds the measured proportion of LDs, with greater overestimation among the more severe cases [14, 22, 29].

Accurate measurement of fat content is required as it is a relevant predictor of liver disease progression [9, 30]. Our study evaluates hepatic steatosis using three diagnostic methods: (I) conventional histology, which is invasive and subjective but has universally accepted cutoffs for grading; (II) DIA, which is invasive and objective; and (III) MRI-PDFF, which is non-invasive and objective. Driven by the limitations of histological scoring systems, DIA has emerged as the new “ground truth” for hepatic steatosis assessment [13, 14]. Actually, the European Medicines Agency (EMA) has recently qualified the first AI tool to analyze biopsy scans in MASLD clinical trials [31]. Our main results are the extremely high correlation between MRI-PDFF and DIA fat fraction. Future studies should now investigate possible grading cut-points for fat fractions (MRI-PDFF and DIA) based on clinical endpoints and not on conventional histology comparison. Of note, only one biopsy sample was obtained per patient, and MRI extracted PDFF after whole-liver segmentation (Supplementary Fig. 7). Although sampling bias is an issue with conventional histology [3, 32], the linearity between DIA and MRI-PDFF suggests that a small proportion of the liver can still provide a representative assessment.

Parallel to previous studies with MR-spectroscopy [33], our study demonstrates a strong correlation between MRI-PDFF and DIA. A progressive increase in the correlation was observed between the size of LDs and PDFF. Conversely, for conventional histology, the strongest correlation was observed with large LDs as pathologists only consider large LDs for grading, disregarding smaller sizes [7, 20, 21]. With DIA, we showed that as the steatosis burden increases, the LDs get larger in size, probably from the fusion of tiny-to-small droplets into larger ones [34]. The significance of quantifying tiny-small LDs has been previously shown as it correlates with more advanced histological features of MASLD and affects the survival of the liver grafts [35, 36].

Certain limitations must be highlighted. First, the calculated sample size was slightly higher than the final recruitment (*n* = 369 vs. 355) due to more exclusions than expected. However, this small difference might not have a significant influence on the results. Second, we used adipophilin to stain steatosis, which is not used in clinical routine. Determining the amount of steatosis on hematoxylin-eosin can be challenging when LDs are very small [5, 21]. For the scope of our study, the use of adipophilin as a specific stain for identifying LD had no added burden of processing [6, 22]. Third, PDFF cutoffs for grading steatosis were based on MASLD studies. These cutoffs were applied to our population of CLD, given that steatotic liver disease can coexist with other etiologies, and including cases without steatosis was also important for the purpose of the study. Fourth, this is a cross-sectional study that did not evaluate the longitudinal assessment of liver fat. Future studies should validate the extent to which longitudinal and parallel liver biopsies and MRI-PDFF correlate with morphological properties of steatosis progression as assessed with DIA.

In conclusion, the DIA-determined size distribution of LDs explains the discrepancies between histology and MRI-PDFF. Compared to histology, PDFF overestimation is due to abundant tiny and small LDs, as MRI captures the whole spectrum of LDs size. Conversely, MRI-PDFF underestimates steatosis in patients with few large LDs, indicating a bias in conventional histology’s subjective visual assessment. These findings challenge the current reference for hepatic steatosis assessment and offer a new interpretation of steatotic liver disease through the implementation of MRI-PDFF in clinical practice.

## Supplementary information

ELECTRONIC SUPPLEMENTARY MATERIAL

## Abbreviations

CLD: Chronic liver disease
DIA: Digital image analysis
H&E: Hematoxylin-eosin
LD: Lipid droplet
MASLD: Metabolic dysfunction-associated steatotic liver disease
MRI: Magnetic resonance imaging
NASH-CRN: Nonalcoholic Steatohepatitis Clinical Research Network system
OR: Odds ratio
PDFF: Proton density fat fraction
VCTE: Vibration-controlled transient elastography
