# Supplementary material for: Digital pathology and lipid droplet size as a key determinant of discrepancies between histology and MRI gradings in steatotic liver disease
Source: Eur Radiol. 2025 Aug 8;36(2):1050–60. doi: 10.1007/s00330-025-11919-0 (PMC12953288; doi:10.1007/s00330-025-11919-0)
Supplement: Supplementary file 1 — ELECTRONIC SUPPLEMENTARY MATERIAL [file 330_2025_11919_MOESM1_ESM.pdf]

Digital Pathology and Lipid Droplet Size as a Key Determinant of Discrepancies between Histology and MRI Gradings in Steatotic Liver Disease

ELECTRONIC SUPPLEMENTARY MATERIAL

Supplementary Material File S1.

Table of Contents

Supplemental methods____2-4.

Supplemental references____4-5.

Supplemental results – text____ 6.

Supplemental results - figures_______________________________________ 7-15.

Supplemental results - tables____ 16-20.

Supplemental material – STARD reporting guidelines_____________________ 21-22.

SUPPLEMENTARY METHODS

Clinical Evaluation

The following characteristics were recorded: age, sex, smoking status, body mass index (BMI), and liver disease etiology. Diagnosis of metabolic dysfunction-associated steatotic liver disease (MASLD) was based on consensus nomenclature including hepatic steatosis identified by ultrasound image, the presence of at least one cardiometabolic risk factor, and alcohol intake below 140g/210g per week in females and males, respectively [1]. MetALD category included those cases with MASLD who consumed increased amounts of alcohol, defined as 140 to 350 g/week and 210 to 420 g/week for females and males, respectively. Cardiometabolic risk factors were defined based on the MASLD consensus [1]: (I) BMI ≥25 kg/m² or waist circumference > 94 cm in male and > 80 cm in female (ethnicity adjusted), (II) fasting glucose ≥100 mg/dL or type 2 diabetes, (III) blood pressure ≥130/85 mmHg or specific antihypertensive drug treatment, (IV) triglycerides ≥150 mg/dL or lipid lowering treatment, (V) HDL-cholesterol ≤40 mg/dL in male and ≤50 mg/dL in female. Diagnosis of autoimmune hepatitis (AIH) was based on biochemical, serological, and histopathological findings in accordance with the simplified criteria of the International AIH Group [2]. Laboratory assessment included glucose, platelets count, bilirubin, albumin, creatinine, AST, ALT, GGT, ferritin, and complete lipid profile.

Vibration-controlled transient elastography (VCTE) examinations were performed with the FibroScan (Echosens, Paris, France) medical device, allowing the quantification of liver stiffness measurement (LSM) and controlled attenuation parameter (CAP). LSM (kPa) and CAP (dB/m) were acquired after ≥4 hours of fasting, using the M or XL probe, as required. VCTE examinations were performed in each center by trained physicians who had previously performed ≥300 determinations. Measurements were acquired from the right liver lobe through an intercostal space. Examinations with at least 10 reliable measurements with >60% success rate and an interquartile range/median value (IQR/M) <30% were considered as valid [3].

Histological evaluation

Tissue slides were evaluated on conventional microscopy by the two central liver pathologists (C.A.C and A.F., >15 years of experience). Histopathological analysis of liver tissue was jointly performed by the two dedicated pathologists, who reviewed the samples together and assigned scores based on mutual consensus. Inter-reader variability between both expert pathologists was not evaluated. Steatosis was graded using hematoxylin-eosin stain. Steatosis grading criterion was consistently applied throughout all different chronic liver diseases, based on the standard frequency of hepatocytes with large LDs displacing the nucleus to the cell periphery (S0: <5%; S1: 5-33%; S2: >33-66%; S3: >66%) [4-6]. For steatotic liver diseases like MASLD or MetALD, the Nonalcoholic Steatohepatitis Clinical Research Network activity score system (NASH-CRN) was used to grade lobular inflammation (0-3), hepatocellular ballooning (0-2) and stage fibrosis on a scale of 0–4 as follows: F0: no fibrosis; F1: perisinusoidal or periportal fibrosis; F2: perisinusoidal and periportal fibrosis; F3: bridging fibrosis; F4: cirrhosis [4]. For chronic hepatitis etiologies like AIH or viral hepatitis, inflammation grade (0-3) and fibrosis stage (0-4) were scored in accordance with the METAVIR scoring system [7]. Fibrosis was staged as follows: F0: no fibrosis; F1: portal fibrosis without septa; F2: portal fibrosis and few septa; F3: numerous septa without cirrhosis; F4: cirrhosis. Significant fibrosis was defined as fibrosis stage ≥F2.

Digital Image Analysis

Stained biopsy slides with adipophilin immunohistochemistry (VITRO Master Diagnostica) were digitized with Ventana iScan HT slide scanner (Roche, Ventana Medical Systems, Inc) which captured whole-slide digital images using a 40x magnification objective and a calibrated camera (4000×4000 pixels being 1mm^2). Adipophilin is a protein that forms the membrane around intracytoplasmic lipid vacuoles and was selected for immunostaining because it offers more precise quantification of LDs and microsteatosis than other fat stains [8,9]. “True” microvesicular steatosis is defined as tiny LDs distending and filling hepatocytes, producing a foamy appearance. They are often not discernible as discrete vacuoles using light microscopy and usually require a fat stain to confirm [10].

The DIA workflow was as follows. After background extraction from the digitalized whole-image and colour normalization to minimize inter-image variability, a custom and automated algorithm based on MATLAB (MATLAB, MathWorks, version R2016a) was implemented for computerized analysis [11]. The algorithm applies color enhancement, shape-based thresholds using contour and edge detection, and structural morphological analysis, to segment LDs and quantify their size. Using adipophilin immunohistochemistry for DIA, tiny LDs were defined as smaller than 1 μm^2 [12]. Furthermore, a 100 μm^2 threshold was used to separate small and large LDs, as a compromise to include pathological large and medium size vacuoles within large LD category of DIA. It has been previously shown that all LDs with a size of 200 μm^2 are able to displace the hepatocyte nucleus [13,14]. However, as the steatosis, activity, fibrosis (SAF) scoring system used by pathologists to grade macrosteatosis quantifies "large or medium-sized lipid droplets" [5], we selected the 100 μm^2 as a size cutoff to help ensure that both medium and large vacuoles would be included within the large LD category of DIA. Using the threshold of 100 μm^2, ≥50% of vacuoles segmented as large LDs are able to displace the hepatocyte nucleus [13]. Using the threshold of 100 μm^2, most vacuoles segmented as large LDs are able to displace the hepatocyte nucleus [13]. Additionally, the vesicle size >100 μm^2 to define macrovesicular steatosis is also supported by other studies using DIA [15].

SUPPLEMENTARY REFERENCES

1. Rinella ME, Lazarus JV, Ratziu V, et al (2023) A multisociety Delphi consensus statement on new fatty liver disease nomenclature. *Hepatology* 78(6):1966-1986.
2. Hennes EM, Zeniya M, Czaja AJ, et al (2008) Simplified criteria for the diagnosis of autoimmune hepatitis. *Hepatology* 48(1):169-176.
3. European Association for the Study of the Liver (2021) EASL Clinical Practice Guidelines on non-invasive tests for evaluation of liver disease severity and prognosis - 2021 update. *J Hepatol* 75(3):659-689.
4. Kleiner DE, Brunt EM, Van Natta M, et al (2005) Design and validation of a histological scoring system for nonalcoholic fatty liver disease. *Hepatology* 41:1313-1321.

5. Bedossa P, Poitou C, Veyrie N, et al (2012) Histopathological algorithm and scoring system for evaluation of liver lesions in morbidly obese patients. *Hepatology* 56(5):1751-1759.
6. Neil DAH, Minervini M, Smith ML, Hubscher SG, Brunt EM, Demetris AJ (2022) Banff consensus recommendations for steatosis assessment in donor livers. *Hepatology* 75(4):1014-1025.
7. Bedossa P, Poynard T (1996) An algorithm for the grading of activity in chronic hepatitis C. The METAVIR Cooperative Study Group. *Hepatology* 24:289-293.
8. Marti-Aguado D, Rodríguez-Ortega A, Mestre-Alagarda C, et al (2021) Digital pathology: accurate technique for quantitative assessment of histological features in metabolic-associated fatty liver disease. *Aliment Pharmacol Ther* 53(1):160–171.
9. Straub BK, Stoeffel P, Heid H, Zimbelmann R, Schirmacher P (2008) Differential pattern of lipid droplet-associated proteins and de novo perilipin expression in hepatocyte steatogenesis. *Hepatology* 47(6):1936-1946.
10. Koenig AB, Tan A, Abdelaal H, Monge F, Younossi ZM, Goodman ZD (2024) Review article: Hepatic steatosis and its associations with acute and chronic liver diseases. *Aliment Pharmacol Ther* 60(2):167-200.
11. Marti-Aguado D, Fernández-Patón M, Alfaro-Cervello C, et al (2021) Digital Pathology Enables Automated and Quantitative Assessment of Inflammatory Activity in Patients with Chronic Liver Disease. *Biomolecules* 11(12):1808.
12. Dempsey JL, Ioannou GN, Carr RM (2023) Mechanisms of Lipid Droplet Accumulation in Steatotic Liver Diseases. *Semin Liver Dis* 43(4):367-382.
13. Nativ NI, Chen AI, Yarmush G, et al (2014) Automated image analysis method for detecting and quantifying macrovesicular steatosis in hematoxylin and eosin-stained histology images of human livers. *Liver Transpl* 20(2):228-236.
14. Gambella A, Salvi M, Molinaro L, et al (2024) Improved assessment of donor liver steatosis using Banff consensus recommendations and deep learning algorithms. *J Hepatol* 80(3):495-504.
15. Windell D, Magness A, Beyer C, et al (2025) AI portal tract detection and characterisation for a regional analysis of steatosis and inflammation in MASLD, MASH, and AIH. *medRxiv* 2025.04.23.25326290.

SUPPLEMENTARY RESULTS

Disagreements between Histology and MRI-PDFF in Cases with Histology-derives Steatosis Grade 1

Among histology-derived S1 (n=69), discordances were observed in 49% (n=34). Of these cases, n=29 was due to MRI-PDFF underestimation while n=5 was due to MRI-PDFF overestimation, as compared to histology. The distribution of LDs size across these categories are represented in the Table.

Digital Image Analysis (DIA)	Discordance: MRI underestimation (n=29)	Concordance (n=35)	Discordance: MRI overestimation (n=5)
• Tiny LD (%)	4.8 ± 2.6%	7.5 ± 2.7%	7.3 ± 3.8%
• Small LD (%)	0.4 ± 0.3%	0.6 ± 0.4%	1.3 ± 0.5%
• Large LD (%)	0.7 ± 0.9%	1.2 ± 1.2%	3.4 ± 3.0%
• Total LDs (%)	5.9 ± 3.5%	9.3 ± 3.1%	12.0 ± 4.6%

The difference in DIA-determined LDs size distribution across categories of agreement between histology and MRI-PDFF were assessed with the one-way analysis of variance, with post hoc Tukey HSD test. Within tiny LD, there were significant differences between concordance category and MRI-PDFF underestimation (P=0.003). Among small LD, there were significant differences between concordance category and MRI-PDFF overestimation (P=0.003). Within large LD, there were significant differences between concordance category and MRI-PDFF overestimation (P=0.007). Among total LDs, there were significant differences between concordance category and MRI underestimation (P=0.002). These results suggest that, in comparison to histology grading, MRI-PDFF underestimation is due to lower steatosis burden. In histology-derived S1, the distribution of LDs size within discordant cases due to MRI-PDFF overestimation were very similar to the ones within concordant cases in histology-derived S2.

SUPPLEMENTARY MATERIAL FIGURES:

Figure 1: Participant flowchart.

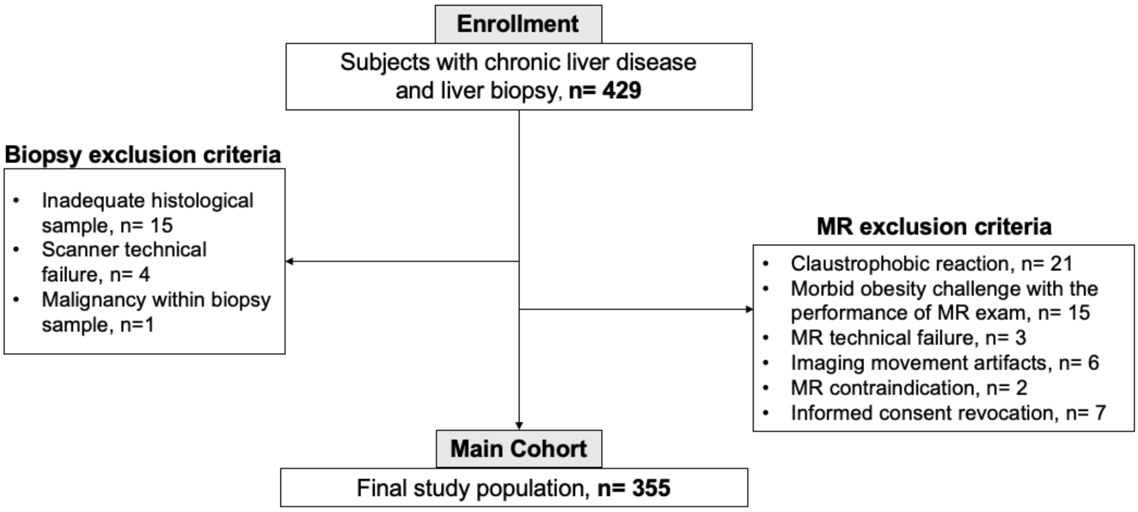

Figure 2: Prevalence (%) of steatosis grades according to histology (dark blue bars) and MRI-PDFF (pink bars). Chi-square test was performed to obtain *P* value.

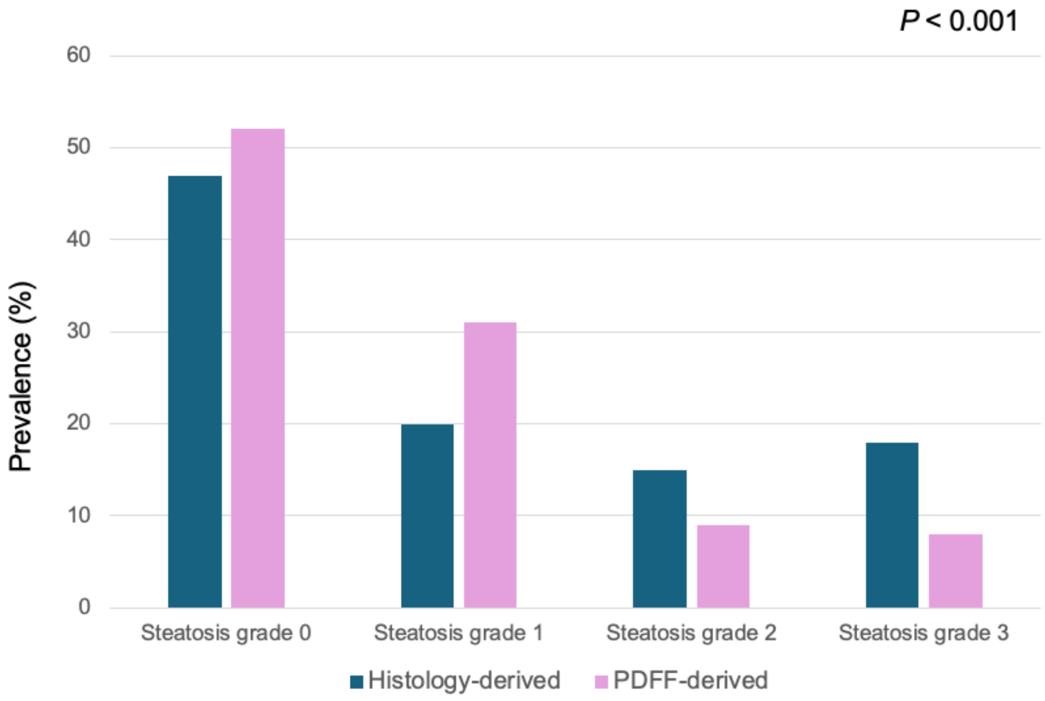

Figure 3: Overview of the distribution of hepatic steatosis grades and the discordances between histology and MRI-PDFF. **(A)** Distribution of PDFF-derived steatosis grades taking histology as reference. **(B)** Distribution of histology-derived steatosis grades taking MRI-PDFF as reference.

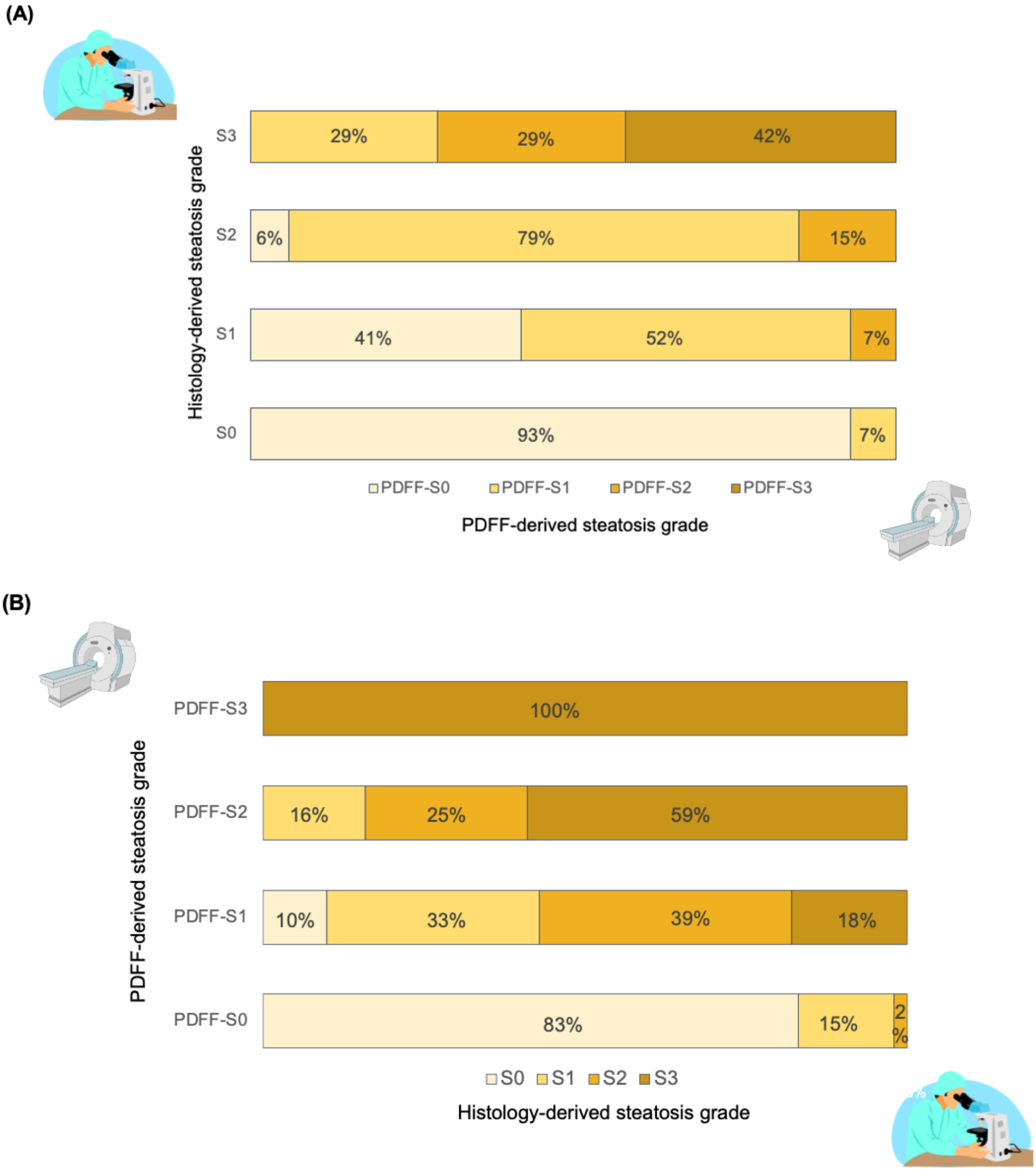

Figure 4: Forest plot summarizing the ratios and confidence intervals of different variables that favors concordance or discordance between histology and MRI-PDFF steatosis gradings.

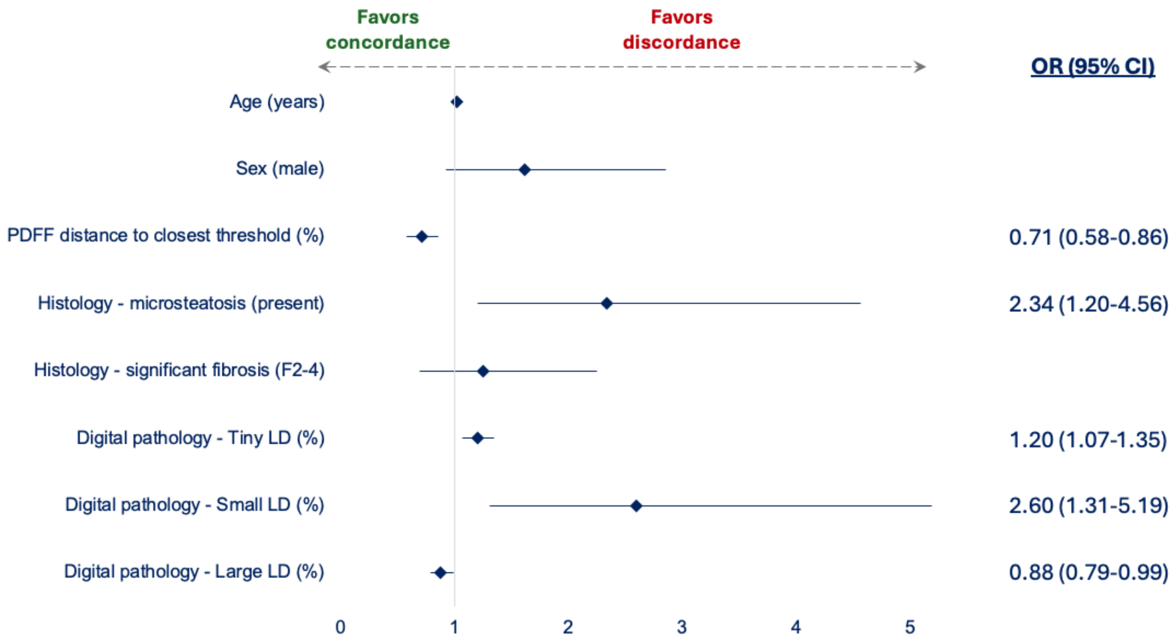

Figure 5: Jitter plot of histology-derived steatosis grade and digital image analysis, categorized according to the size of lipid droplets (LDs); **(A)** tiny LD proportionate area, **(B)** small LD proportionate area, **(C)** large LD proportionate area, and **(D)** total LDs proportionate area. Spearman rank correlation coefficient [ρ] is shown for each linear regression. The strongest correlation was seen between large LD proportionate area and histology-derived steatosis grade.

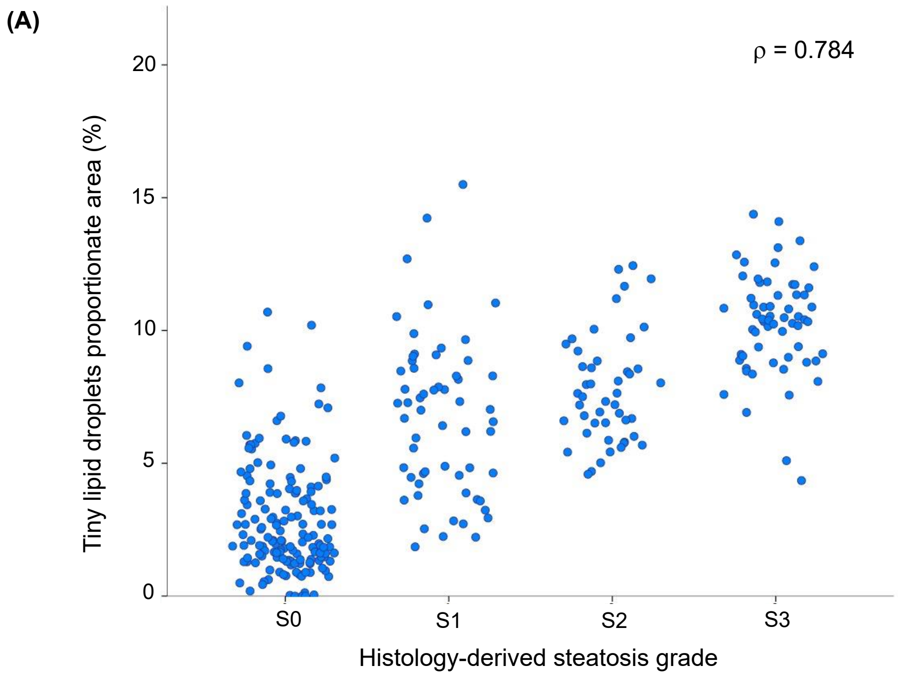

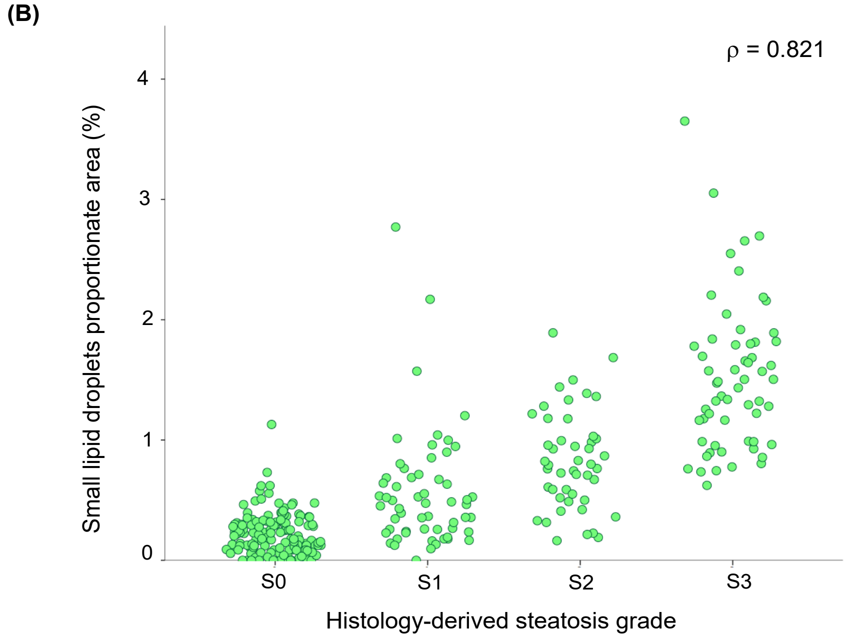

(C)

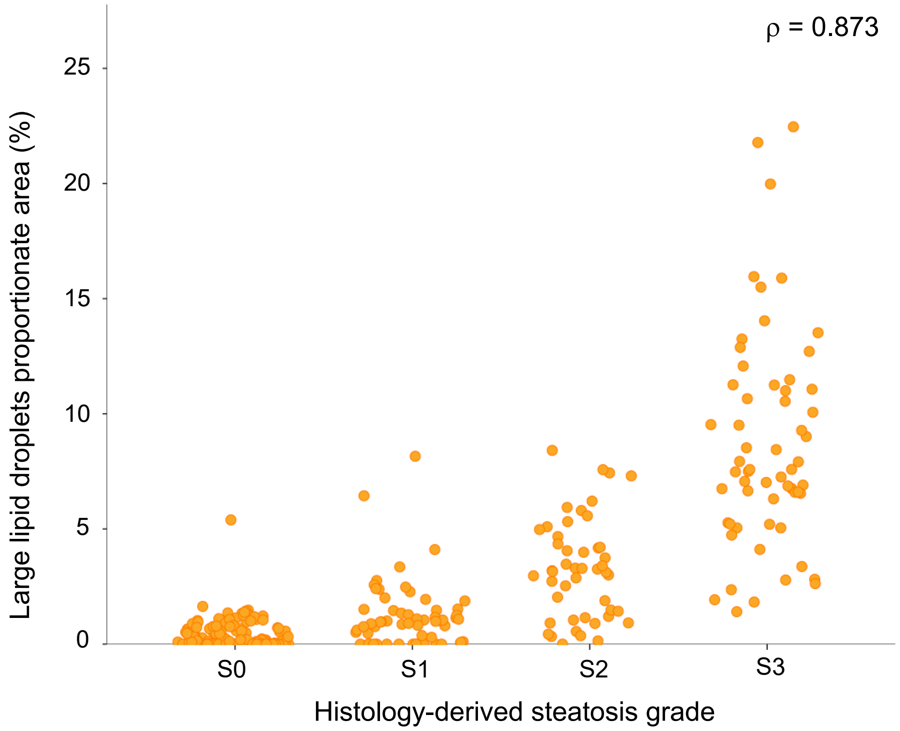
ρ = 0.873
25
20
15
10
5
0
Large lipid droplets proportionate area (%)
S0
S1
S2
S3
Histology-derived steatosis grade

(D)

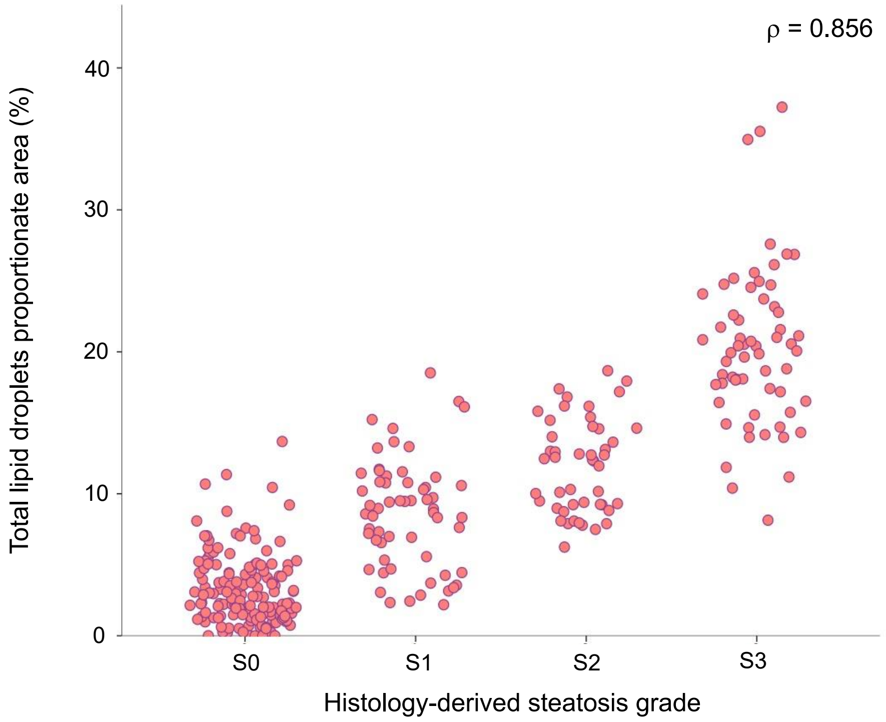
ρ = 0.856
40
30
20
10
0
Total lipid droplets proportionate area (%)
S0
S1
S2
S3
Histology-derived steatosis grade

Figure 6: Scatterplot of MRI-PDFF and digital image analysis, categorized according to the size of lipid droplets (LDs); **(A)** tiny LD proportionate area, **(B)** small LD proportionate area, **(C)** large LD proportionate area, and **(D)** total LDs proportionate area. Pearson's correlation coefficient [r] is shown for each linear regression. The gray dotted line represents the linear regression fit. The strongest correlation was seen between total LDs proportionate area and MRI-PDFF.

(A)

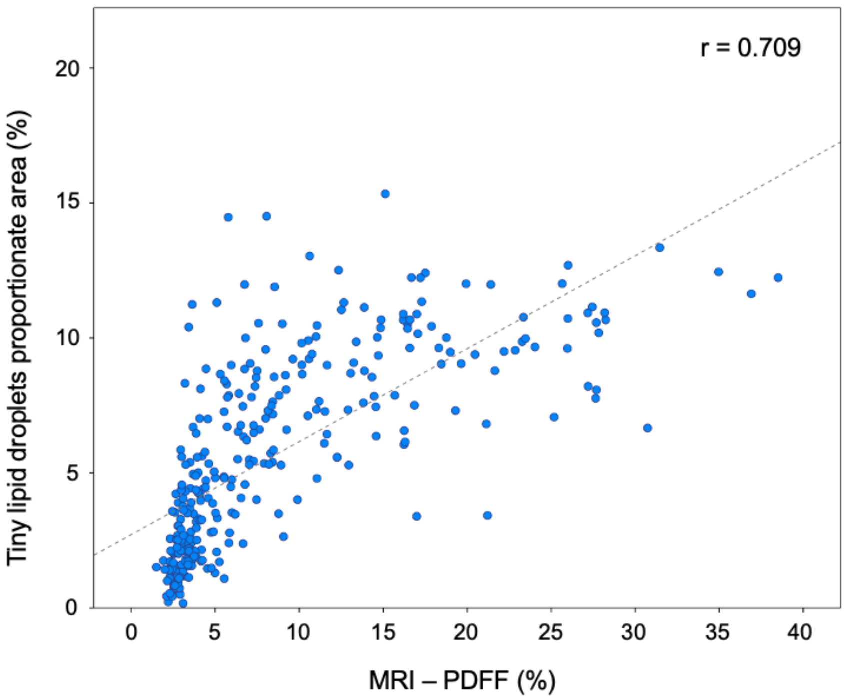

(B)

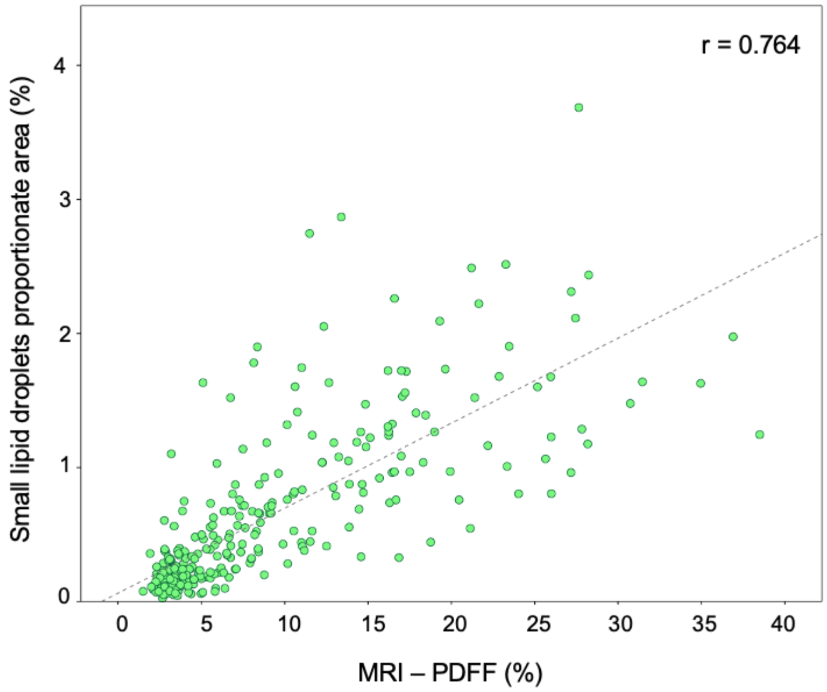

(C)

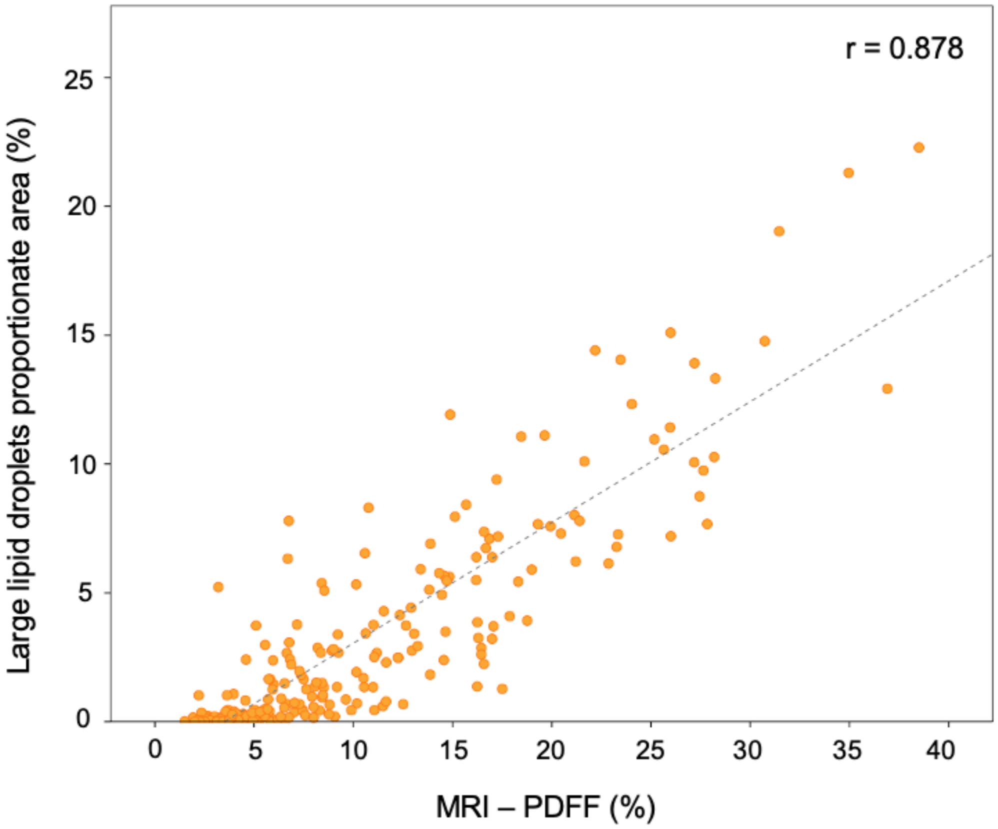
r = 0.878
Large lipid droplets proportionate area (%)
MRI – PDFF (%)
0
5
10
15
20
25
0
5
10
15
20
25
30
35
40

(D)

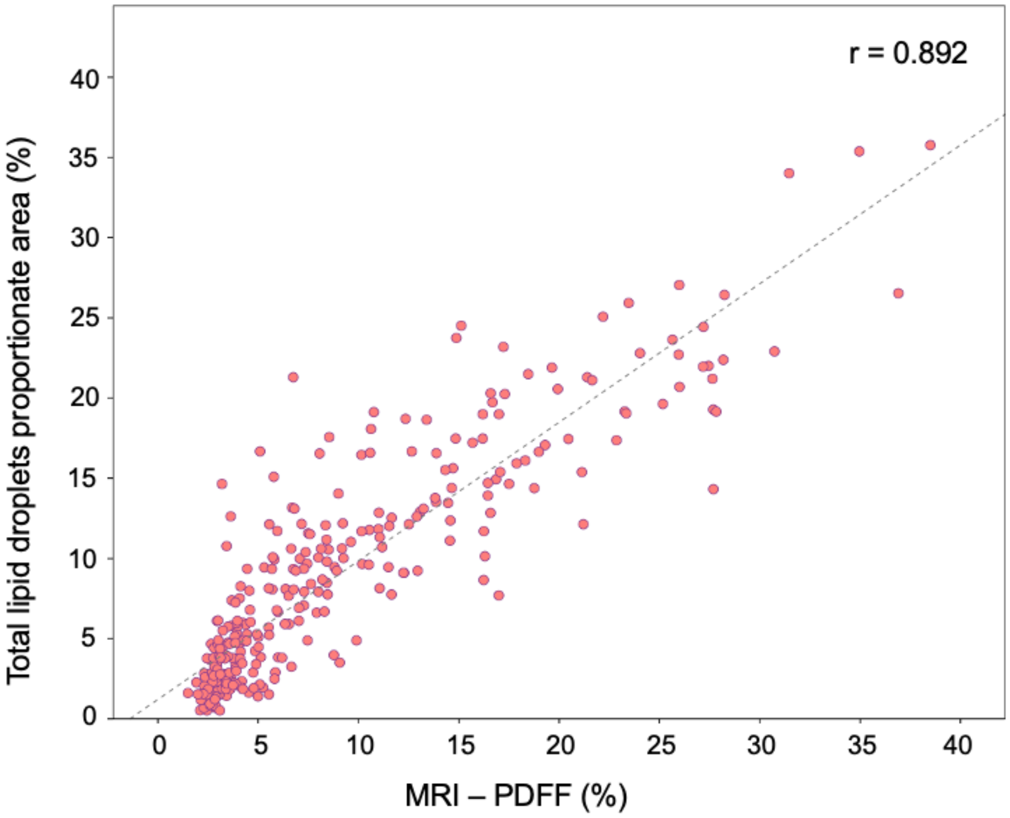
r = 0.892
Total lipid droplets proportionate area (%)
MRI – PDFF (%)
0
5
10
15
20
25
30
35
40
0
5
10
15
20
25
30
35
40

Figure 7: Schematic overview of MRI whole liver segmentation analysis and liver biopsy sampling bias. MRI analysis used a whole liver parenchyma segmentation approach (estimated volume: 1000 cm^3) while liver biopsy only evaluates a minimal proportion of the liver (0.05 cm^3, 1 in 20.000 parts).

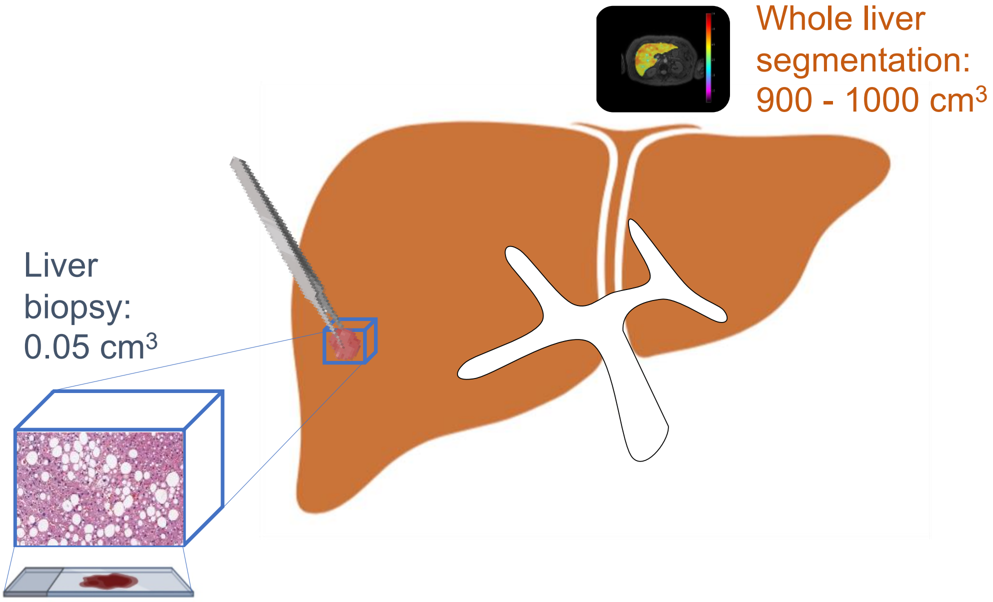

SUPPLEMENTARY TABLES

Supplementary Table 1: Baseline histological features

Characteristic	Overall Sample
Biopsy length (mm)	20 (16-23)
Number of portal tracts	10 (7-13)
Hepatocellular ballooning	
• Grade 0	208 (58%)
• Grade 1	104 (30%)
• Grade 2	43 (12%)
Inflammation grade	
• Grade 0	100 (28%)
• Grade 1	164 (46%)
• Grade 2	75 (21%)
• Grade 3	16 (5%)
Mallory's hyaline	33 (9%)
Fibrosis stage	
• Stage 0	124 (35%)
• Stage 1	78 (22%)
• Stage 2	73 (20%)
• Stage 3	48 (14%)
• Stage 4	32 (9%)

Note. —Unless otherwise specified, data are numbers of participants (n=355), with percentages in parentheses. Data are reported as medians with interquartile ranges.

Supplementary Table 2: Distribution of the total lipid droplets (LDs) proportionate area (%) by DIA across steatosis grades derived by histology vs. MRI-PDFF.

Steatosis grade	Total LDs (%) across histology-derived grades	Total LDs (%) across MRI-PDFF-derived grades	*p-value*
• Grade 0	S0: 3.3 ± 2.3%	PDFF-S0: 3.6 ± 2.7%	0.336
• Grade 1	S1: 8.3 ± 3.8%	PDFF-S1: 10.9 ± 4.4%	<0.001
• Grade 2	S2: 11.7 ± 3.2%	PDFF-S2: 16.2 ± 3.9%	<0.001
• Grade 3	S3: 20.0 ± 5.4%	PDFF-S3: 23.4 ± 5.1%	0.007

Note. —Data are reported as means ± standard deviations. Comparisons between paired distributions were assessed with the Wilcoxon matched pairs test stratified by steatosis grades.
Abbreviations. —DIA, digital image analysis; LDs, lipid droplets; MRI, Magnetic Resonance Imaging; PDFF, Proton Density Fat Fraction; PDFF-S, PDFF determined steatosis grades S, histological steatosis grades.

Supplementary Table 3: Distribution of clinical characteristics and analytical parameters in concordant *vs*. discordant cases between histology- and PDFF-derived steatosis grade.

Variables	Concordance (n=228)	Discordance (n=127)	*p-value*
Sex (female)	143 (63%)	66 (52%)	0.048
Age (years)	53 (46- 62)	58 (48- 65)	0.003
Smoking status (current/former)	55 (25%)	32 (26%)	0.764
BMI (kg/m^2)	26.9 ± 4.9	29.8 ± 5.1	<0.001
MASLD etiology	110 (48%)	109 (85%)	<0.001
Obesity	60 (27%)	55 (44%)	0.001
Arterial hypertension	69 (31%)	67 (54%)	<0.001
Diabetes mellitus	38 (17%)	55 (44%)	<0.001
Dyslipidemia	96 (43%)	79 (64%)	<0.001
Hypothyroidism	32 (17%)	23 (24%)	0.204
VCTE - CAP (dB/m)	256 ± 66	299 ± 56	<0.001
VCTE – LSM (kPa)	8.8 ± 7.3	11.7 ± 9.9	0.003
Glucose (mg/dL)	95 (86-107)	106 (94-130)	<0.001
Platelet count (×10^9/L)	230 ± 71	229 ± 72	0.946
Creatinine (mg/dL)	0.78 ± 0.21	0.84 ± 0.29	0.046
ALT (U/L)	57 (35-112)	50 (32-72)	0.082
AST (U/L)	42 (31-80)	41 (32-56)	0.259
GGT (U/L)	90 (47-182)	84 (46-229)	0.709
Total bilirubin (mg/dL)	0.6 (0.5-0.9)	0.6 (0.5-0.9)	0.585
Albumin (g/dL)	4.4 (4.1-4.5)	4.4 (4.2-4.7)	0.022
Ferritin (mg/mL)	103 (44-215)	136 (48-265)	0.158
Triglycerides (mg/dL)	105 (73-149)	139 (94-191)	<0.001
Total cholesterol (mg/dL)	194 ± 42	183 ± 40	0.011
Low-density lipoprotein (mg/dL)	118 ± 35	109 ± 36	0.014
High-density lipoprotein (mg/dL)	56 (45-70)	49 (42-61)	<0.001

Note. —Unless otherwise specified, data are numbers of participants (n=355), with percentages in parentheses. Data are reported as means ± standard deviations when normally distributed and medians with interquartile ranges when the distribution is skewed. Differences in characteristics across groups were tested using independent t-test or Mann–Whitney U test for continuous variables and the chi-square test for categorical variables.

Abbreviations. —ALT, Alanine aminotransferase; AST, Aspartate aminotransferase; BMI, body mass index; CAP, controlled attenuation parameter; GGT, g-glutamyl transferase; LSM, liver stiffness measurement; VCTE, vibration controlled transient elastography.

Supplementary Table 4: Distribution of MRI-PDFF across histology-derived steatosis grades, stratified by the presence of microsteatosis as determined by pathologists.

Histological grade	Microsteatosis not present (n=206)	Microsteatosis present (n=149)	*p-value*
• S0	3.5 ± 1.3%	4.7 ± 1.9%	0.008
• S1	5.4 ± 1.8%	8.2 ± 3.9%	0.001
• S2	11.5 ± 3.0%	11.3 ± 4.4%	0.862
• S3	18.7 ± 4.9%	20.3 ± 7.1%	0.589

Note. —Data are reported as means ± standard deviations. Comparisons between paired distributions were assessed with the Wilcoxon matched pairs test stratified by histological grades.
Abbreviations. — MRI, Magnetic Resonance Imaging; PDFF, Proton Density Fat Fraction; S, histological steatosis grades.

Supplementary Table 5: Distribution of MRI-PDFF and DIA-derived total lipid droplets proportionate area, across histology-derived steatosis grades. Similar values for both methods were obtained in all histology-derived steatosis grades except for S0.

Histological grade	MRI-PDFF (%)	DIA-LDs (%)	*p-value*
• S0	3.5 ± 1.4%	3.3 ± 2.3%	0.239
• S1	7.2 ± 3.5%	8.3 ± 3.7%	0.071
• S2	11.3 ± 4.0%	11.7 ± 3.2%	0.629
• S3	20.2 ± 7.0%	20.0 ± 5.3%	0.860

Note. —Data are reported as means ± standard deviations. Comparisons between paired distributions were assessed with the Wilcoxon matched pairs test stratified by histological grades.
Abbreviations. —DIA, digital image analysis; LDs, lipid droplets; MRI, Magnetic Resonance Imaging; PDFF, Proton Density Fat Fraction; S, histological steatosis grades.

STARD – 2015 checklist

Section & Topic	No	Item	Reported on page #
**TITLE OR ABSTRACT**			
	**1**	Identification as a study of diagnostic accuracy using at least one measure of accuracy (such as sensitivity, specificity, predictive values, or AUC)	2
**ABSTRACT**			
	**2**	Structured summary of study design, methods, results, and conclusions (for specific guidance, see STARD for Abstracts)	2
**INTRODUCTION**			
	**3**	Scientific and clinical background, including the intended use and clinical role of the index test	4
	**4**	Study objectives and hypotheses	4, 5
**METHODS**			
Study design	**5**	Whether data collection was planned before the index test and reference standard were performed (prospective study) or after (retrospective)	6
Participants	**6**	Eligibility criteria	6
	**7**	On what basis potentially eligible participants were identified (such as symptoms, results from previous tests, inclusion in registry)	6
	**8**	Where and when potentially eligible participants were identified (setting, location and dates)	6
	**9**	Whether participants formed a consecutive, random or convenience series	6
Test methods	**10a**	Index test, in sufficient detail to allow replication	6, 7
	**10b**	Reference standard, in sufficient detail to allow replication	6, 7
	**11**	Rationale for choosing the reference standard (if alternatives exist)	4, 6, 7
	**12a**	Definition of and rationale for test positivity cut-offs or result categories of the index test, distinguishing pre-specified from exploratory	6
	**12b**	Definition of and rationale for test positivity cut-offs or result categories of the reference standard, distinguishing pre-specified from exploratory	7
	**13a**	Whether clinical information and reference standard results were available to the performers/readers of the index test	6
	**13b**	Whether clinical information and index test results were available to the assessors of the reference standard	7
Analysis	**14**	Methods for estimating or comparing measures of diagnostic accuracy	8, 9
	**15**	How indeterminate index test or reference standard results were handled	NA
	**16**	How missing data on the index test and reference standard were handled	NA
	**17**	Any analyses of variability in diagnostic accuracy, distinguishing pre-specified from exploratory	8, 9
	**18**	Intended sample size and how it was determined	8
**RESULTS**			
Participants	**19**	Flow of participants, using a diagram	Supp Fig. 1
	**20**	Baseline demographic and clinical characteristics of participants	Table 1

	**21a**	Distribution of severity of disease in those with the target condition	10, Table 1
	**21b**	Distribution of alternative diagnoses in those without the target condition	Table 1
	**22**	Time interval and any clinical interventions between index test and reference standard	10
Test results	**23**	Cross tabulation of the index test results (or their distribution) by the results of the reference standard	Figure 5
	**24**	Estimates of diagnostic accuracy and their precision (such as 95% confidence intervals)	Table 2
	**25**	Any adverse events from performing the index test or the reference standard	N/A
**DISCUSSION**			
	**26**	Study limitations, including sources of potential bias, statistical uncertainty, and generalisability	15
	**27**	Implications for practice, including the intended use and clinical role of the index test	13, 14
**OTHER INFORMATION**			
	**28**	Registration number and name of registry	6
	**29**	Where the full study protocol can be accessed	Title page
	**30**	Sources of funding and other support; role of funders	Title page
